# WBM-DLNets: Wrapper-Based Metaheuristic Deep Learning Networks Feature Optimization for Enhancing Brain Tumor Detection

**DOI:** 10.3390/bioengineering10040475

**Published:** 2023-04-14

**Authors:** Muhammad Umair Ali, Shaik Javeed Hussain, Amad Zafar, Muhammad Raheel Bhutta, Seung Won Lee

**Affiliations:** 1 Department of Intelligent Mechatronics Engineering, Sejong University, Seoul 05006, Republic of Korea; umair@sejong.ac.kr (M.U.A.); amad@sejong.ac.kr (A.Z.); 2 Department of Electrical and Electronics, Global College of Engineering and Technology, Muscat 112, Oman; s.javeedhussain@gcet.edu.om; 3 Department of Electrical and Computer Engineering, University of UTAH Asia Campus, Incheon 21985, Republic of Korea; 4 Department of Precision Medicine, Sungkyunkwan University School of Medicine, Suwon 16419, Republic of Korea

**Keywords:** brain MRI, deep learning networks, wrapper-based metaheuristic algorithms, brain tumor detection, image processing

## Abstract

This study presents wrapper-based metaheuristic deep learning networks (WBM-DLNets) feature optimization algorithms for brain tumor diagnosis using magnetic resonance imaging. Herein, 16 pretrained deep learning networks are used to compute the features. Eight metaheuristic optimization algorithms, namely, the marine predator algorithm, atom search optimization algorithm (ASOA), Harris hawks optimization algorithm, butterfly optimization algorithm, whale optimization algorithm, grey wolf optimization algorithm (GWOA), bat algorithm, and firefly algorithm, are used to evaluate the classification performance using a support vector machine (SVM)-based cost function. A deep-learning network selection approach is applied to determine the best deep-learning network. Finally, all deep features of the best deep learning networks are concatenated to train the SVM model. The proposed WBM-DLNets approach is validated based on an available online dataset. The results reveal that the classification accuracy is significantly improved by utilizing the features selected using WBM-DLNets relative to those obtained using the full set of deep features. DenseNet-201-GWOA and EfficientNet-b0-ASOA yield the best results, with a classification accuracy of 95.7%. Additionally, the results of the WBM-DLNets approach are compared with those reported in the literature.

## 1. Introduction

Uncontrolled cell development causes brain abnormalities, such as brain tumors. This syndrome has been associated with a considerable number of deaths worldwide and is commonly recognized as a lethal disease [1]. Brain tumors, whether benign or malignant, cause damage to adjacent brain tissue owing to the increased pressure exerted on the skull [2]. In 2022, approximately 88,970 people suffered brain tumors, among which 63,040 were benign, whereas the remaining 25,930 were malignant [3,4]. Gliomas, meningiomas, and pituitary gland tumors are several types of tumors that may affect the human brain [5,6]. Hence, the early detection of brain tumors is critical for timely and efficient treatment.

Neuro-oncologists can effortlessly and rapidly diagnose brain tumors using computer-aided diagnostic (CAD) technologies. The development of new imaging technologies, such as magnetic resonance imaging (MRI), to visualize the characteristics of brain tumors has increased in recent years. MRI is a useful technique that offers detailed scans of tissues and organs. Owing to its noninvasive nature, MRI is the most reliable method for locating and measuring brain tumors [7]. Correctly analyzing multidimensional MRI data can assist in localizing and tracking disease development and advising on treatment. These scans provide vital information regarding the size, shape, and location of brain tumors without exposing the patient to significant levels of ionizing radiation [8]. Radiologists use MRIs to assess and treat brain tumors. Hence, a major concern in radiology is the assessment of brain tumors using imaging techniques for various brain lesions. Several significant obstacles must be overcome to manage brain tumors, including early identification and therapy, which are crucial for patient survival.

In healthcare imaging, machine learning has been utilized for disease diagnosis in breast [9,10], brain [11,12,13], and lung [14,15] tumors. Recently, research on brain tumor segmentation, with numerous segmentation methods using different datasets, has increased considerably. Currently, three types of segmentation models have been developed [16]: supervised machine learning [17], clustering-based segmentation [18,19], and deep learning [13].

The segmentation challenge was reformulated using supervised learning algorithms as a tumor pixel classification problem. This approach produces the desired segmentation classes as the output of the model after feeding the retrieved features of the image as the input. A previous study [17] presented a hybrid random forest and active contour model for brain tumor classification. Another study used support vector machine (SVM) to categorize brain tumors [20,21]. Both the aforementioned studies utilized brain MRI to detect brain tumors. Owing to the similarity of the brain MRI scans, the precision of the model was low for the subcategorization of brain tumors.

Clustering-based segmentation methods divide MRI scans into distinct subcategories and pinpoint the area of interest in each scan. In this method, pixels with a high degree of similarity within each region are categorized as belonging to a particular region. By contrast, pixels that differ from those inside an area are classified as regions that do not belong to the area of interest. K-means clustering is a popular unsupervised machine-learning approach for separating an area of interest from other components of an image. It has been applied for segmenting brain tumors with fair precision as it uses a modest amount of processing time and power [22], and is most effective when large datasets are used. Its flaws include susceptibility to outliers and inadequate definition of the tumor region [23]. In one study, the authors extracted KAZE and speeded up robust local-level features to classify brain MRIs into glioma, meningioma, no tumor, and pituitary classes [18]. Subsequently, they used 8 × 8 pixels for feature extraction, and applied k-means clustering to segment 400 features for each descriptor. The model yielded an accuracy of 95.33% for the multiclass problems. In a subsequent study [19], the authors applied the PSO-based ReliefF algorithm to remove redundant features. They reduced the feature vector from 800 to 169 and achieved an accuracy of 96.30% using the k-fold method. In another study, the tumor area was compared with other brain imaging areas [24] and the expected tumor parts could not be distinguished with certainty. Clustering may lead to imprecise tumor size identification, resulting in ineffective therapy and higher morbidity and fatality rates.

Deep learning segmentation models accurately calculate the features of brain MRI images using the layers of a deep learning network [25]. A deep learning network was developed to segment complete and masked brain MRI images into two categories [26]. The proposed model had classification accuracies of 92.9% and 89.5% for a complete MRI and a mask, respectively. Abiwinanda et al. [27] presented a simplified deep learning network architecture to categorize MRI images into subgroups; the accuracy of this model was only 84.19%. In another study, a 22-layer deep learning network exhibited an accuracy of 95.56% for tertiary class problems (glioma, meningioma, and pituitary gland tumor) [28]. Subsequently, the authors applied data augmentation to balance the data; nevertheless, the use of data augmentation in real-time applications was unreliable. A 25-layer deep learning network reported an accuracy of 92.66% [29]. Pretrained networks, such as GoogleNet and ResNet-50, also reported classification accuracies of 98% and 97.2% for brain tumor detection, respectively [30,31]. Training a pretrained model requires considerable time. To address this problem, a study computed deep features using pretrained networks and utilized them to train conventional classifiers [32]. The concatenated deep features of ShuffleNet V2, DenseNet-169, and MnasNet yielded an accuracy of 93.72% using the SVM model. However, reportedly, the training time of the model was prolonged because of the large size of the training feature vectors. Therefore, the feature removal technique can be applied to improve model accuracy. Dokeroglu et al. [33] recently examined different wrapper-based feature-selection approaches. Wrapper techniques investigate the performance of each feature subset and combine a metaheuristic optimization algorithm with a classifier.

Therefore, in this study, wrapper-based metaheuristic deep-learning network (WBM-DLNet) feature optimization approaches were investigated to improve the classification accuracy of brain MRI scans. First, brain MRI scans were preprocessed to resize and reduce noise. Various pretrained models, such as DarkNet-19, DarkNet-53, DenseNet-201, EfficientNet-b0, GoogLeNet365, GoogLeNet, Inception-ResNet-v2, Inception-v3, MobileNet-v2, NASNet-Mobile, ResNet-101, ResNet-50, ResNet-18, ShuffleNet, SqueezeNet, and Xception, were used to extract information from brain MRI images. Wrapper approaches with various metaheuristic optimization algorithms, such as the marine predators algorithm (MPA), atom search optimization algorithm (ASOA), Harris hawks optimization algorithm (HHOA), butterfly optimization algorithm (BOA), whale optimization algorithm (WOA), grey wolf optimization algorithm (GWOA), bat algorithm (BA), and firefly algorithm (FA), were used to examine the classification performance using SVM. An empirical approach was used to select the best network. Based on the results of various feature subsets, a new concatenated feature vector was formed to evaluate the performance. The performance of the proposed framework was evaluated using an online brain MRI dataset.

## 2. Methods and Materials

### 2.1. Brain MRI Dataset

In this study, a collection of brain MRI images available online was used. The dataset utilized herein was retrieved from the Kaggle website [34] and comprised four brain MRI classes. Table 1 presents information on the brain MRI dataset.

### 2.2. Preprocessing

Preprocessing is required to eliminate unnecessary information and reduce noise from brain MRI scans. Cropping is typically employed for removing nonbrain regions and reducing noise. In this study, a cropping approach was employed to compute the extreme points of the brain area. Dilation and erosion processes, the two fundamental morphological processes, were applied to reduce noise. In an image, dilation involves adding pixels to object borders, whereas erosion involves removing pixels from object boundaries. The size and shape of the structuring element used to process the image determine the number of pixels added to or subtracted from the objects. Additional information on the preprocessing can be found in [32,35]. In addition, the preprocessed images were scaled to meet the input requirements of the pretrained networks employed in this study.

### 2.3. Deep Feature Extraction

**Figure 1:**
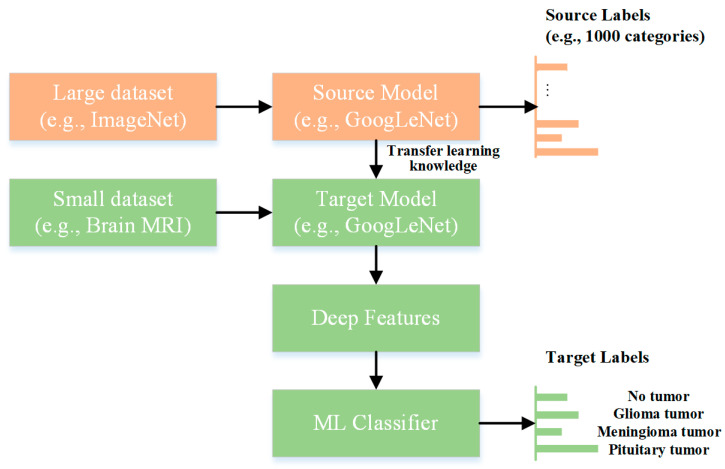
Deep feature extraction using GoogLeNet.

Features are the primary factors when categorizing images. Identifying essential features is vital for improving classification performance. Although brain MRI feature extraction can be performed manually or using a deep learning network, manual extraction is very time-consuming. The considerable diversity in brain MRI scans determines their accuracy. By contrast, deep learning neural networks employ a combination of convolutional, pooling, and fully connected layers to build the model. Pretrained deep learning models can extract deep features using the transfer learning method when the dataset is sufficiently small. Figure 1 illustrates the concept of deep feature extraction using a trained model (GoogLeNet).

Deep neural networks that have been trained on extensive image classification tasks are known as pre-trained deep learning models and are capable of extracting hierarchical features from images. These deep features are acquired by passing an image through the network and analyzing the activation of one or more layers, thus indicating the response of the network to various image features. These activations may be used to categorize or compare images and feed them into other classical machine learning models, such as SVM.

DarkNet-19, DarkNet-53, DenseNet-201, EfficientNet-b0, GoogLeNet365, GoogLeNet, Inception-ResNet-v2, Inception-v3, MobileNet-v2, NASNet-Mobile, ResNet-101, ResNet-50, ResNet-18, ShuffleNet, SqueezeNet, and Xception were the prominent pre-trained CNN models used in this study. Each of these models contains distinguishing features that capture multiple aspects of images, such as local and global patterns or fine-grained details. These models can be used in a range of computer vision applications, such as object identification, image segmentation, and image retrieval.

### 2.4. Wrapper-Based Feature Selection Approach

Feature selection is essential in numerous machine-learning applications because it affects model accuracy. Therefore, relevant features must be utilized to effectively characterize and classify items. However, the inclusion of irrelevant features may lead to low accuracy. Therefore, the most valuable features must be determined to increase the classification accuracy of the model. During the feature-selection procedure in this study, a subset of a wider set of features was selected to build the machine learning model. Note that a specific criterion is used to assess the quality of the new subset [36]. This can be accomplished using various strategies, such as filter-based, wrapper-based, or embedded feature selection [37]. These approaches can also assist in minimizing model complexity, thus resulting in quicker and more efficient processing.

**Figure 2:**
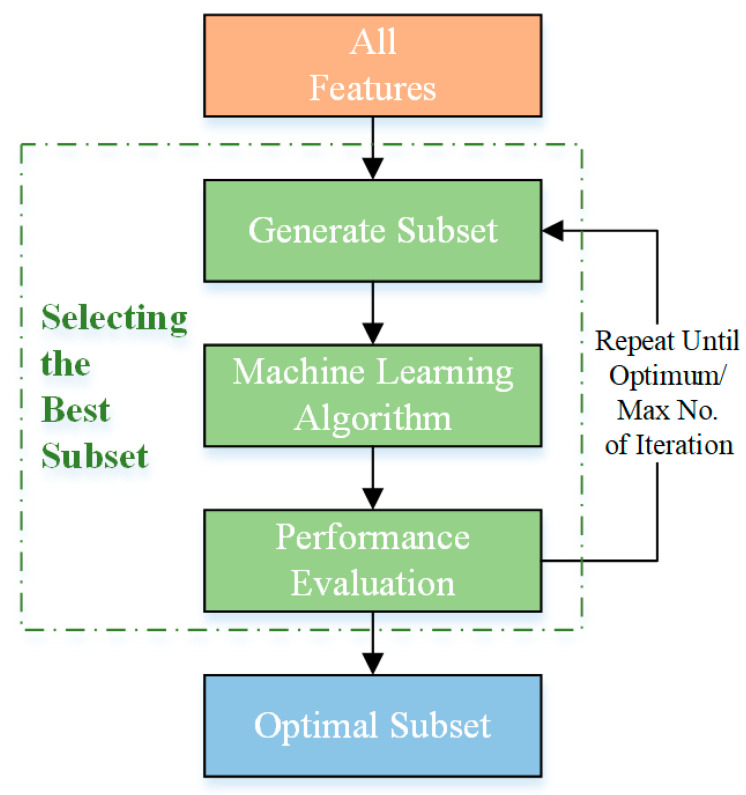
Working flow for the wrapper-based approach.

In this study, wrapper-based algorithms were used to select the most appropriate features for training a machine learning model. Wrapper algorithms are machine learning methods for evaluating the performance of a group of features when used with a particular model (the “wrapper”) [38]. The goal of the wrapper is to assess the impact of the selected features on the accuracy of the model. The wrapper algorithm either selects the current subset of features or seeks an improved subset based on the evaluation results. This procedure is repeated until an optimal subset of features is obtained. Figure 2 illustrates the concept of the wrapper approach. For optimal feature selection, this study employed a variety of metaheuristic algorithms and wrapper-based approaches.

The goal of metaheuristic techniques is to estimate the solutions to complex problems. They are referred to as “meta” because they integrate multiple low-level heuristics to manage high-level optimization tasks [39]. Examples of metaheuristic algorithms include MPA, ASOA, HHOA, BOA, WOA, GWOA, BA, and FA [33,40]. In wrapper feature selection methods, learning algorithms evaluate the performance of the resulting feature subsets during classification. Metaheuristics are used as search algorithms to identify new optimal subsets [41]. The cost functions of all optimization methods are specified in Equation (1).

(1) $$\min(I)=\omega (1-\mathrm{Accuracy})+\sigma \left(\frac{\mathrm{number}\;\mathrm{of}\;\mathrm{selected}\;\mathrm{features}}{\mathrm{Total}\;\mathrm{number}\;\mathrm{of}\;\mathrm{features}}\right)$$

where, the value of ω and σ are 0.99 and 0.01, respectively [42].

#### 2.4.1. Marine Predators Algorithm (MPA)

MPA is an optimization technique inspired by nature that adheres to principles that naturally control the best foraging tactics and encounter rates between predators and prey in marine environments. The hunting and foraging strategies of marine predators, such as sharks and dolphins, serve as the basis for MPA [43,44]. The four key phases used for the simulation are predation, reproduction, migration, and exploration. During the predation phase, the algorithm hunts for and catches food; during the reproduction phase, it transfers genetic material to the progeny; during the exploration phase, it searches for new locations in the search space; and during the migration phase, it relocates the predators to new locations.

Numerous optimization problems, including feature selection, image segmentation, and data clustering, have been effectively solved using this technique. MPA is very efficient and helpful in addressing optimization issues in various fields because it can obtain global optima in complicated search spaces [45].

#### 2.4.2. Atom Search Optimization Algorithm (ASOA)

The principle of ASOA is the mobility and interaction of atoms in a physical system [46]. It is a population-based method in which a group of atoms searches for the best answer in a predetermined search space, and it involves the four key steps of initialization, attraction, repulsion, and migration. During the initialization phase, this method creates a collection of atoms with different positions and velocities in the search space; during the attraction stage, the atoms travel toward the best answer; during the repulsion stage, they move away from one another to explore other regions of the search space; and during the migration step, they relocate to a new location in the search space. Numerous optimization issues have been successfully solved using ASOA [47].

#### 2.4.3. Harris Hawks Optimization Algorithm (HHOA)

Bairathi and Gopalani proposed the optimization technique of HHOA, which is based on the cooperative hunting style of Harris hawks [48]. This algorithm is built on a group of individuals called “hawks” who stand in for potential solutions. The four key phases of HHOA are scouting, prey finding, trap setting, and attacks. During the scouting phase, the program randomly creates an initial population of hawks; during the prey-finding phase, the hawks explore the search space to determine the best answers; during the trap-setting phase, they corner the victim; and during the attack phase, they work together to capture it. Numerous optimization issues have been solved successfully using the HHOA [49].

#### 2.4.4. Butterfly Optimization Algorithm (BOA)

The metaheuristic optimization BOA was developed by Arora and Singh after they studied the feeding habits of butterflies in the environment [50]. The BOA mimics butterfly motion and behavior to explore and adapt to changing search space conditions. It has five stages: initialization, search, selection, mutation, and update. During the initialization stage, this technique randomly creates an initial butterfly population; during the search stage, the butterflies explore the search space and identify the best solutions; during the selection stage, they select the best answers from the population; during the mutation stage, the algorithm introduces random modifications to the selected solutions to explore new search space regions; and during the update stage, the algorithm changes the population, based on the latest solutions discovered during the mutation stage. The BOA has been used to solve various complex engineering problems [51].

#### 2.4.5. Whale Optimization Algorithm (WOA)

Mirjalili and Lewis [52] proposed the WOA in 2016, the WOA is based on the hunting behavior of humpback whales, which hunt in groups and employ bubble nets to catch tiny fish or krill. The WOA algorithm can solve complex optimization problems by discovering the prey, enclosing it, and moving it in spiral bubble-net patterns. The process begins with a random solution. Subsequently, other agents alter their locations by selecting the best search agent and selecting a target for attack, which may be the best search agent or a random whale. The WOA has been successfully used for various optimization issues, including function optimization, scheduling, and data clustering [33].

#### 2.4.6. Grey Wolf Optimization Algorithm (GWOA)

Mirjalili et al. developed the GWOA in 2014 based on the grey wolf social structure and hunting skills [53]. Grey wolves hunt in packs, each headed by an alpha wolf, the dominant member of the crew. The group’s second leader, the beta wolf, assists the alpha wolf and conveys the instructions, and the subordinate or delta wolves support. When the prey is within a certain range, the pack surrounds and assaults it. Alpha, beta, and delta wolves are the top three GWOA algorithmic solutions. The remaining wolves are omega wolves and have no bearing on the decisions made in the subsequent iterations. The GWOA employs this social structure and prey-hunting strategies as a mathematical representation to solve optimization issues in domains ranging from computer science to engineering, mathematics, physics, and finance [54,55].

#### 2.4.7. Bat Algorithm (BA)

Yang first presented the BA in 2010 [56] as an optimization technique. This approach is modeled after how bats search for prey by flying randomly, making noise, and listening to echoes [57]. The BA maintains a population of alternative solutions to the optimization problem. It iteratively enhances these solutions using random walk and exploitation procedures. The exploitation operation enables the algorithm to focus on the most promising answers. By contrast, the random-walk operation mimics the unpredictable search behavior of bats. The algorithm also uses a frequency-tuning mechanism motivated by the frequency modulation of bat sounds, which allows it to break out of local optima and discover superior solutions. In addition, the BA is easy to deploy and does not require complicated parameter tuning. Echolocation can be coded as a method for improving the objective function [58].

#### 2.4.8. Firefly Algorithm (FA)

Motivated by the flashing qualities of fireflies, Yang presented the FA in 2010 [59], based on flashes, which attract potential mates and scare away predators. In FA, flashing features are established and used as functions to address combinatorial optimization problems [60]. Fireflies attract fellow flies and move toward brighter fireflies based on their brightness levels. The greater the separation between fireflies, the less enticing they appear to be. In the absence of a brighter light, fireflies travel randomly. Consequently, the intensity of light and appeal levels have key influences on the FA. A specific function can be used to alter brightness at a specific location. Attraction is determined by other fireflies because it is based on the distance and absorption coefficient.

## 3. Proposed WBM-DLNets Framework for Brain Tumor Detection

MRI is the most reliable tool used by radiologists for the detection of brain tumors. This study aimed to build an intelligent model that can detect brain tumors and classify them into subcategories (glioma, meningioma, and pituitary gland tumor).

**Figure 3:**
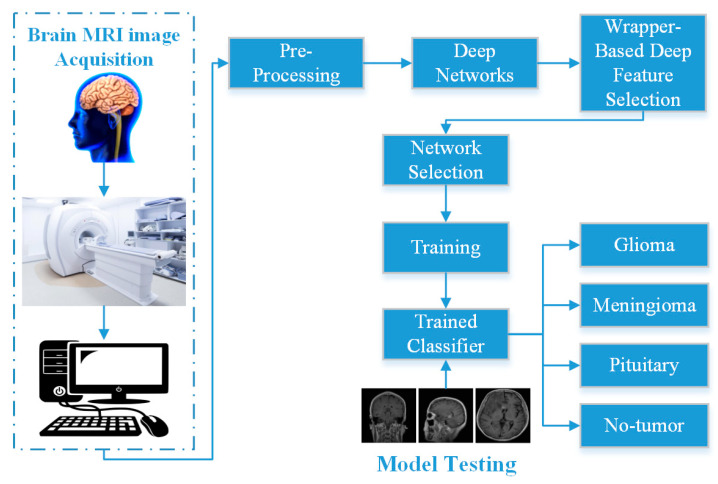
Block diagram of the proposed WBM-DLNets.

**Figure 4:**
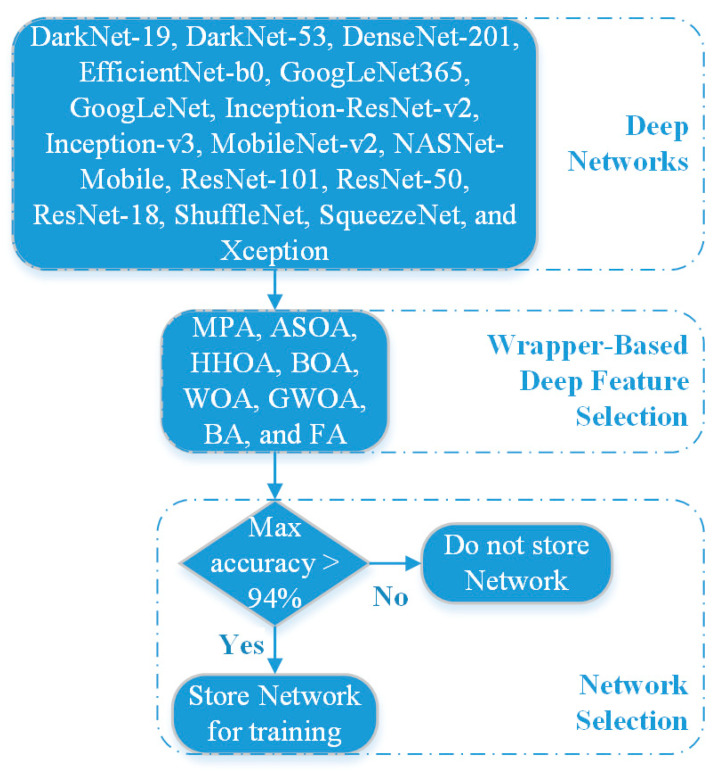
Network selection framework.

As mentioned in Section 2.2, herein, the input MRIs were preprocessed to eliminate noise and irrelevant information. Next, the pre-trained networks were used to calculate the deep features. The most relevant features were retrieved using MPA-, ASOA-, HHOA-, BOA-, WOA-, GWOA-, BA-, and FA-wrapper-based feature selection techniques. A block diagram of the proposed WBM-DLNets brain tumor detection approach and a flow chart of network selection are shown in Figure 3 and Figure 4, respectively.

In the network selection phase, the proposed approach selects deep learning networks with a metaheuristic optimizer having a maximum accuracy of more than 94% for the deep features of an individual deep learning network. Herein, MATLAB’s “Jx-WFST,” a Jxwrapper feature selection library, was used to implement all methods (https://www.mathworks.com/matlabcentral/fileexchange/84139-wrapper-feature-selection-toolbox, accessed on 7 February 2023). Further, the SVM model was used for brain MRI classification [61,62].

## 4. Results and Discussion

In this study, the WBM-DLNet feature optimization technique was applied to enhance the classification accuracy of brain tumor detection. An online brain tumor classification dataset was used to test the presented WBM-DLNets feature optimization technique [34]. Discrimination between the MRI images of the subcategories of tumors was accomplished by utilizing the deep features of various pre-trained deep learning networks, as discussed above. The WBM algorithms discussed above (MPA, ASOA, HHOA, BOA, WOA, GWOA, BA, and FA) were used to extract valuable information. Further, MATLAB 2022b, operating on a personal computer with the following specifications, was used for all processing and analysis: Core i7, 12th Generation, 32 GB of RAM, NVIDIA GeForce RTX 3050, 1 TB SSD, and 64-bit Windows 11. The extracted feature subset was classified using an SVM and 0.2-holdout validation technique. The parameters of each algorithm are listed in Table 2.

**Figure 5:**
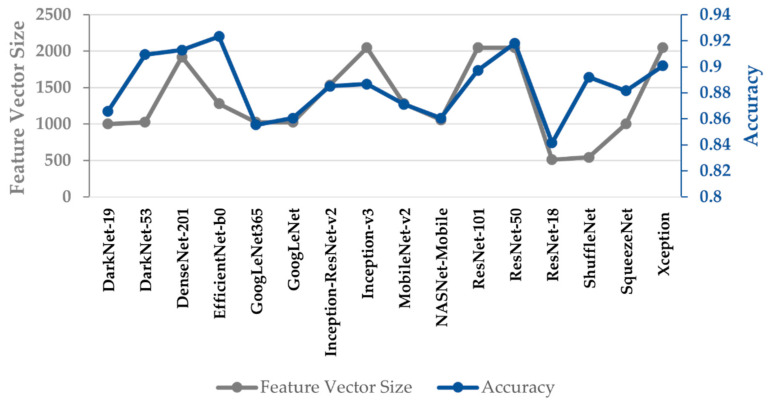
Performance of the SVM classifier trained with full deep features of pre-trained models.

For each brain MRI image, the deep features of the various pretrained networks were extracted before the SoftMax layer. The initial rate, number of epochs, and momentum were 0.001, 100, and 0.9, respectively. The results of the full deep features of various pretrained networks are presented as classification accuracy and feature vector size in Figure 5.

A thorough analysis of the results presented in Figure 4 reveals that the SVM trained using the deep features of DarkNet-53, DenseNet-201, EfficientNet-b0, ResNet-50, and Xception deep-learning networks yielded classification accuracies above 90% for brain MRI images, and the feature vector sizes of these pretrained networks were 1024, 1920, 1280, 2048, and 2048, respectively. EfficientNet-b0 exhibited a classification rate of 92.33% with a training feature vector size of 1280 for brain tumor detection. The results of the various metaheuristic algorithms used to determine the optimal deep features of the various pretrained networks are presented in Table 3. The detailed results of all networks and optimization algorithms in terms of accuracy, feature vector size, and processing time are shown in Figure S1 in the Supplementary Materials.

**Figure 6:**
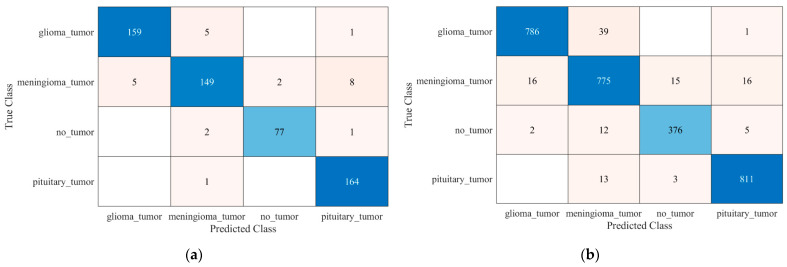
Confusion matrix of the proposed WBM-DLNets for brain tumor detection: (**a**) 0.2-holdout; (**b**) five-fold cross-validation.

As presented in Table 3, all optimization algorithms significantly increased and decreased the accuracy and feature vector size, respectively. However, only two deep learning networks (DenseNet-201 and EfficientNet-b0) yielded accuracies greater than 94%. DenseNet-201 with GWOA exhibited almost 3.31% higher accuracy relative to the full features of DenseNet-201 (1920 features). Additionally, it used three times fewer features to train the SVM model (651 features). In the case of EfficientNet-b0, three optimization algorithms, MPA, ASOA, and WOA, classified the brain MRI images with 94.4%, 94.9%, and 94.4% accuracy, respectively. Based on the network selection criteria discussed in Section 3 and Figure 4, the proposed approach selected only the deep features of the EfficientNet-b0-ASOA. The deep features of DenseNet-201-GWOA and EfficientNet-b0-ASOA were concatenated to train the SVM model using the 0.2-holdout and five-fold cross-validation techniques. The confusion metrics of the WBM-DLNets are shown in Figure 6. The results were thoroughly analyzed using the true positive rate (TPR), false negative rate (FNR), positive predictive value (PPV), and false discovery rate (FDR) of the developed machine learning model, as presented in Table 4. Equation (2) can be used to compute TPR, FNR, PPV, FDR, and accuracy.

(2) $$\begin{array}{c} \mathrm{TPR}=\frac{\mathrm{True}\;\mathrm{positive}}{\mathrm{No}.\;\mathrm{of}\;\mathrm{real}\;\mathrm{positive}}\\ \mathrm{FNR}=\frac{\mathrm{False}\;\mathrm{negative}}{\mathrm{No}.\;\mathrm{of}\;\mathrm{real}\;\mathrm{positive}}\\ \mathrm{PPV}=\frac{\mathrm{True}\;\mathrm{positive}}{\mathrm{True}\;\mathrm{positive}+\mathrm{False}\;\mathrm{positive}}\\ \mathrm{FDR}=\frac{\mathrm{False}\;\mathrm{positive}}{\mathrm{True}\;\mathrm{positive}+\mathrm{False}\;\mathrm{positive}}\\ \mathrm{Accuracy}\;(\%)=\frac{\mathrm{No}.\;\mathrm{of}\;\mathrm{correctly}\;\mathrm{classified}\;\mathrm{images}}{\mathrm{Total}\;\mathrm{no}.\;\mathrm{of}\;\mathrm{images}}\times 100 \end{array}\}$$

In the case of holdout validation, 159 of the 165 MRI images of glioma tumors were correctly classified and had a TPR of 96.4% (Figure 6a and Table 4). The no-tumor class contained 80 MRI images to test the model. A total of 77 brain MRI images with a PPV of 97.5% were classified correctly. The proposed WBM-DLNets achieved a high classification accuracy of 95.6% with only 1292 feature vector sizes for holdout validation. In the five-fold cross-validation, the proposed model slightly increased the classification accuracy, as presented in Figure 6b and Table 4. The model correctly predicted 811 out of 824 pituitary gland tumor class MRI images with a TPR of 98.1%.

**Figure 7:**
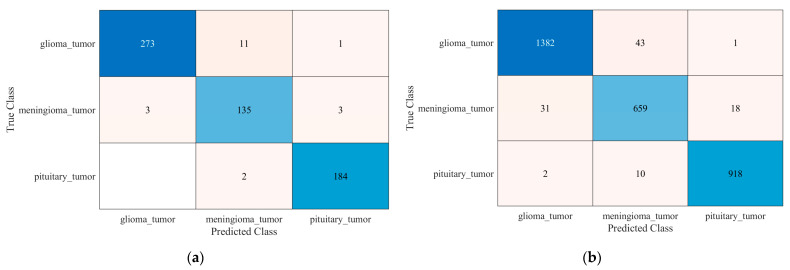
Confusion matrix of the proposed WBM-DLNets for brain tumor detection for another dataset [63]: (**a**) 0.2-holdout; (**b**) five-fold cross-validation.

Another brain MRI dataset comprising 233 patients was used to validate the adaptability and accuracy [63]. A total of 3064 brain MRI scans were obtained at two hospitals in China (Nanfang Hospital and General Hospital), of which 1426 images comprised glioma tumors, 708 comprised meningioma tumors, and 930 comprised pituitary gland tumors [64]. The results of the proposed approach for the new dataset are presented in Figure 7 and Table 5.

The results presented in Figure 7 and Table 5 demonstrate the adaptability and high classification performance of the proposed approach. As presented in Table 6, the results of the proposed WBM-DLNets approach were also compared with those reported in the latest literature.

Recently, the use of machine learning techniques in medical imaging for diagnostic applications has increased. Several researchers have investigated various learning techniques for detecting brain tumors using MRI images [28,29,32,65].

Deep learning networks have a very high level of accuracy in classifying brain MRI images [28,29]. Irmak [29] proposed a brain tumor detection CNN model that yielded an accuracy of 92.66% for subclassification. Additionally, a pretrained deep learning network has been utilized to detect brain tumors [65]; however, the model training it is too time-consuming. Another study [32] concatenated the deep features of various pre-trained models and achieved 93.72% accuracy. The feature vector was excessively large and contained various redundant features. Filter-based feature reduction approaches have also been reported to enhance classification performance [18]. Filtering strategies select features based on their applicability to the dependent variables. However, they do not consider how they affect the model performance. By contrast, wrapper approaches test the effectiveness of a feature subset by training the model with them. Occasionally, a threshold value must be selected to remove unnecessary data in filter-based methods. Wrapper-based strategies have been shown to be more accurate in classifying data. Therefore, in this study, a WBM-DLNet feature optimization approach was proposed to improve the classification performance of brain tumor detection. The WBM-DLNet feature optimization approach and previously published studies are compared in Table 6. The findings revealed that the proposed WBM-DLNet feature optimization technique outperforms competing methods in terms of classification rate. The proposed brain tumor detection approach may help physicians identify tumors quickly and effectively.

## 5. Conclusions

In this study, WBM-DLNet feature optimization algorithms were utilized to enhance brain tumor detection classification performance. The deep features of the 16 pretrained deep learning networks were computed. Eight metaheuristic optimization algorithms (MPA, ASOA, HHOA, BOA, WOA, GWOA, BA, and FA) were applied to determine the optimal deep features of all networks using the SVM-based cost function. All metaheuristic optimization algorithms significantly enhanced the classification performance and reduced the feature vector size of each pretrained model. Subsequently, a deep-learning network selection approach was applied to determine the best deep features. The best deep features were concatenated to train the SVM model. The model exhibited the highest classification rate of 95.7% with DenseNet-201-GWOA and EfficientNet-b0-ASOA deep feature-trained SVM models. The model was further validated using a new dataset and exhibited a high classification performance of 96.7%. Therefore, the proposed WBM-DLNet feature optimization algorithm can be considered useful for automatic brain tumor detection.

## Figures and Tables

**Table 1:** Details of online brain MRI dataset.

	Glioma Tumor	Meningioma Tumor	No Tumor	Pituitary Tumor
Brain MRI images	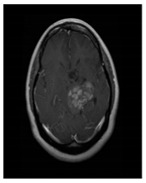	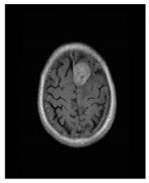	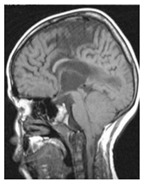	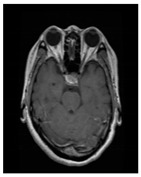
No. of images per class	826	822	395	827

**Table 2:** Parameters for each algorithm.

MPA	ASOA	HHOA	BOA	WOA	GWOA	BA	FA
Number of iterations = 50	Number of iterations = 50	Number of iterations = 50	Number of iterations = 50	Number of iterations = 50	Number of iterations = 50	Number of iterations = 50	Number of iterations = 50
Population size = 10	Population size = 10	Population size = 10	Population size = 10	Population size = 10	Population size = 10	Population size = 10	Population size = 10
Fish aggregating devices effect = 0.2	Depth weight = 50	Levy component = 1.5	Modular modality = 0.01	Constant = 1		Maximum frequency = 2	Absorption coefficient = 1
Constant = 0.5	Multiplier weight = 0.2		Switch probability = 0.8			Minimum frequency = 0	Constant = 1
Levy component = 1.5						Constant = 0.9	Light amplitude =1
						Maximum loudness = 2	Control alpha = 0.97
						Maximum pulse rate = 1	

**Table 3:** Classification performance of each pre-trained network with metaheuristic algorithms for brain tumor detection.

Network	MPA		ASOA		HHOA		BOA		WOA		GWOA		BA		FA	
	Accuracy	Feature Vector Size	Accuracy	Feature Vector Size	Accuracy	Feature Vector Size	Accuracy	Feature Vector Size	Accuracy	Feature Vector Size	Accuracy	Feature Vector Size	Accuracy	Feature Vector Size	Accuracy	Feature Vector Size
DarkNet-19	0.906	267	0.895	516	0.894	172	0.880	413	0.895	304	0.909	303	0.894	494	0.892	447
DarkNet-53	0.930	732	0.922	504	0.923	575	0.913	481	0.918	712	0.934	362	0.913	491	0.920	521
DenseNet-201	0.930	939	0.916	979	0.922	1270	0.894	912	0.909	970	0.946	651	0.908	964	0.918	950
EfficientNet-b0	0.944	821	0.949	641	0.939	862	0.925	551	0.944	654	0.939	451	0.934	669	0.936	621
GoogLeNet365	0.894	388	0.901	499	0.885	487	0.878	472	0.882	542	0.899	401	0.887	522	0.887	479
GoogLeNet	0.889	667	0.873	531	0.883	501	0.852	521	0.873	690	0.895	338	0.864	518	0.871	493
Inception-ResNet-v2	0.915	533	0.909	753	0.908	739	0.909	802	0.902	782	0.925	488	0.904	754	0.908	763
Inception-v3	0.904	878	0.911	997	0.908	1023	0.895	764	0.895	1174	0.923	768	0.901	990	0.895	966
MobileNet-v2	0.913	812	0.902	647	0.890	770	0.883	623	0.887	738	0.894	512	0.892	659	0.897	633
NASNet-Mobile	0.885	575	0.869	505	0.871	714	0.864	461	0.873	854	0.887	364	0.866	497	0.871	549
ResNet-101	0.925	1279	0.927	1057	0.915	1231	0.923	927	0.915	1536	0.927	826	0.913	985	0.911	1005
ResNet-50	0.934	1228	0.937	1013	0.939	1254	0.916	877	0.932	1068	0.939	692	0.927	1017	0.934	1016
ResNet-18	0.878	274	0.876	242	0.875	315	0.854	214	0.864	428	0.887	210	0.880	242	0.873	233
ShuffleNet	0.916	217	0.904	274	0.911	374	0.887	271	0.895	321	0.918	218	0.894	286	0.901	256
SqueezeNet	0.904	519	0.913	499	0.902	619	0.885	516	0.894	852	0.901	385	0.889	485	0.890	483
Xception	0.920	809	0.929	1035	0.916	1149	0.908	889	0.911	978	0.930	775	0.916	1002	0.915	1009

**Table 4:** Results of WBM-DLNets for tumor detection.

Validation	Class	TPR (%)	FNR (%)	PPV (%)	FDR (%)	Accuracy (%)
0.2-holdout	glioma_tumor	96.4	3.6	97.0	3.0	95.6
	meningioma_tumor	90.9	9.2	94.9	5.1	
	no_tumor	96.3	3.8	97.5	2.5	
	pituitary_tumor	99.4	0.6	94.3	5.7	
Five-fold cross-validation	glioma_tumor	95.2	4.8	97.8	2.2	95.7
	meningioma_tumor	94.3	5.7	92.4	7.6	
	no_tumor	95.2	4.8	95.4	4.6	
	pituitary_tumor	98.1	1.9	97.4	2.6	

**Table 5:** Results of WBM-DLNets for tumor detection for another dataset [63].

Validation	Class	TPR (%)	FNR (%)	PPV (%)	FDR (%)	Accuracy (%)
0.2-holdout	glioma_tumor	95.8	4.2	98.9	1.1	96.7
	meningioma_tumor	95.7	4.3	91.2	8.8	
	pituitary_tumor	98.9	1.1	97.9	2.1	
Five-fold cross-validation	glioma_tumor	96.9	3.1	97.7	2.3	96.6
	meningioma_tumor	93.1	6.9	92.6	7.4	
	pituitary_tumor	98.7	1.3	98.0	2.0	

**Table 6:** Comparison of the proposed WBM-DLNets with other studies.

Reference	Accuracy (%)
Almalki et al. [18]	95.33
Abiwinanda et al. [27]	84.19
Irmak [29]	92.66
Kang et al. [32]	93.72
Rehman et al. [65]	95.86
WBM-DLNets (Proposed)	95.7 and 96.7

## Data Availability

The data used to support the findings of this study are included in the article.

## Supplementary Materials

The following supporting information can be downloaded at: https://www.mdpi.com/article/10.3390/bioengineering10040475/s1, Figure S1: Detailed results of all the networks and the optimization algorithms in terms of accuracy, feature vector size, and processing time: (a) MPA; (b) ASOA; (c) HHOA; (d) BOA; (e) WOA; (f) GWOA; (g) BA; (h) FA.

## References

[1] Louis D.N., Perry A., Reifenberger G., von Deimling A., Figarella-Branger D., Cavenee W.K., Ohgaki H., Wiestler O.D., Kleihues P., Ellison D.W. (2016). The 2016 World Health Organization Classification of Tumors of the Central Nervous System: A summary. Acta Neuropathol..

[2] Haj-Hosseini N., Milos P., Hildesjö C., Hallbeck M., Richter J., Wårdell K. Fluorescence spectroscopy and optical coherence tomography for brain tumor detection. Proceedings of the SPIE Photonics Europe, Biophotonics: Photonic Solutions for Better Health Care.

[3] Ren W., Hasanzade Bashkandi A., Afshar Jahanshahi J., Qasim Mohammad AlHamad A., Javaheri D., Mohammadi M. (2023). Brain tumor diagnosis using a step-by-step methodology based on courtship learning-based water strider algorithm. Biomed. Signal Process. Control.

[4] Brain Tumor Facts. https://braintumor.org/brain-tumors/about-brain-tumors/brain-tumor-facts/#:~:text=Today%2C%20an%20estimated%20700%2C000%20people,will%20be%20diagnosed%20in%202022.

[5] American Cancer Society www.cancer.org/cancer.html.

[6] American Society of Clinical Oncology https://www.cancer.net/cancer-types/brain-tumor/diagnosis.

[7] Wulandari A., Sigit R., Bachtiar M.M. Brain tumor segmentation to calculate percentage tumor using MRI. Proceedings of the 2018 International Electronics Symposium on Knowledge Creation and Intelligent Computing (IES-KCIC).

[8] Xu Z., Sheykhahmad F.R., Ghadimi N., Razmjooy N. (2020). Computer-aided diagnosis of skin cancer based on soft computing techniques. Open Med..

[9] Kebede S.R., Debelee T.G., Schwenker F., Yohannes D. (2020). Classifier Based Breast Cancer Segmentation. J. Biomim. Biomater. Biomed. Eng..

[10] Debelee T.G., Amirian M., Ibenthal A., Palm G., Schwenker F. Classification of Mammograms Using Convolutional Neural Network Based Feature Extraction. Proceedings of the Information and Communication Technology for Development for Africa.

[11] Gab Allah A.M., Sarhan A.M., Elshennawy N.M. (2021). Classification of Brain MRI Tumor Images Based on Deep Learning PGGAN Augmentation. Diagnostics.

[12] Alanazi M.F., Ali M.U., Hussain S.J., Zafar A., Mohatram M., Irfan M., AlRuwaili R., Alruwaili M., Ali N.H., Albarrak A.M. (2022). Brain Tumor/Mass Classification Framework Using Magnetic-Resonance-Imaging-Based Isolated and Developed Transfer Deep-Learning Model. Sensors.

[13] Almalki Y.E., Ali M.U., Kallu K.D., Masud M., Zafar A., Alduraibi S.K., Irfan M., Basha M.A.A., Alshamrani H.A., Alduraibi A.K. (2022). Isolated Convolutional-Neural-Network-Based Deep-Feature Extraction for Brain Tumor Classification Using Shallow Classifier. Diagnostics.

[14] Debelee T.G., Kebede S.R., Schwenker F., Shewarega Z.M. (2020). Deep Learning in Selected Cancers’ Image Analysis—A Survey. J. Imaging.

[15] Pandian R., Vedanarayanan V., Ravi Kumar D.N.S., Rajakumar R. (2022). Detection and classification of lung cancer using CNN and Google net. Meas. Sens..

[16] Allah AM G., Sarhan A.M., Elshennawy N.M. (2023). Edge U-Net: Brain tumor segmentation using MRI based on deep U-Net model with boundary information. Expert Syst. Appl..

[17] Ma C., Luo G., Wang K. (2018). Concatenated and Connected Random Forests with Multiscale Patch Driven Active Contour Model for Automated Brain Tumor Segmentation of MR Images. IEEE Trans. Med. Imaging.

[18] Almalki Y.E., Ali M.U., Ahmed W., Kallu K.D., Zafar A., Alduraibi S.K., Irfan M., Basha M.A.A., Alshamrani H.A., Alduraibi A.K. (2022). Robust Gaussian and Nonlinear Hybrid Invariant Clustered Features Aided Approach for Speeded Brain Tumor Diagnosis. Life.

[19] Ali M.U., Kallu K.D., Masood H., Hussain S.J., Ullah S., Byun J.H., Zafar A., Kim K.S. (2022). A Robust Computer-Aided Automated Brain Tumor Diagnosis Approach Using PSO-ReliefF Optimized Gaussian and Non-Linear Feature Space. Life.

[20] Kumari R. (2013). SVM classification an approach on detecting abnormality in brain MRI images. Int. J. Eng. Res. Appl..

[21] Ayachi R., Ben Amor N. Brain tumor segmentation using support vector machines. Proceedings of the Symbolic and Quantitative Approaches to Reasoning with Uncertainty: 10th European Conference, ECSQARU 2009.

[22] Almahfud M.A., Setyawan R., Sari C.A., Setiadi D.R.I.M., Rachmawanto E.H. An Effective MRI Brain Image Segmentation using Joint Clustering (K-Means and Fuzzy C-Means). Proceedings of the 2018 International Seminar on Research of Information Technology and Intelligent Systems (ISRITI).

[23] Abdel-Maksoud E., Elmogy M., Al-Awadi R. (2015). Brain tumor segmentation based on a hybrid clustering technique. Egypt. Inform. J..

[24] Kaya I.E., Pehlivanlı A.Ç., Sekizkardeş E.G., Ibrikci T. (2017). PCA based clustering for brain tumor segmentation of T1w MRI images. Comput. Methods Programs Biomed..

[25] Nazir M., Shakil S., Khurshid K. (2021). Role of deep learning in brain tumor detection and classification (2015 to 2020): A review. Comput. Med. Imaging Graph..

[26] Pereira S., Meier R., Alves V., Reyes M., Silva C.A. (2018). Automatic Brain Tumor Grading from MRI Data Using Convolutional Neural Networks and Quality Assessment.

[27] Abiwinanda N., Hanif M., Hesaputra S.T., Handayani A., Mengko T.R. (2019). Brain Tumor Classification Using Convolutional Neural Network.

[28] Badža M.M., Barjaktarović M.Č. (2020). Classification of Brain Tumors from MRI Images Using a Convolutional Neural Network. Appl. Sci..

[29] Irmak E. (2021). Multi-Classification of Brain Tumor MRI Images Using Deep Convolutional Neural Network with Fully Optimized Framework. Iran. J. Sci. Technol. Trans. Electr. Eng..

[30] Deepak S., Ameer P.M. (2019). Brain tumor classification using deep CNN features via transfer learning. Comput. Biol. Med..

[31] Çinar A., Yildirim M. (2020). Detection of tumors on brain MRI images using the hybrid convolutional neural network architecture. Med. Hypotheses.

[32] Kang J., Ullah Z., Gwak J. (2021). MRI-Based Brain Tumor Classification Using Ensemble of Deep Features and Machine Learning Classifiers. Sensors.

[33] Dokeroglu T., Deniz A., Kiziloz H.E. (2022). A comprehensive survey on recent metaheuristics for feature selection. Neurocomputing.

[34] Chakrabarty N., Kanchan S. Brain Tumor Classification (MRI). https://www.kaggle.com/datasets/sartajbhuvaji/brain-tumor-classification-mri?select=Training.

[35] Rosebrock A. Finding extreme points in contours with Open CV. https://www.pyimagesearch.com/2016/04/11/finding-extreme-points-in-contours-with-opencv/.

[36] Dash M., Liu H. (1997). Feature selection for classification. Intell. Data Anal..

[37] Guyon I., Elisseeff A. (2003). An introduction to variable and feature selection. J. Mach. Learn. Res..

[38] Kohavi R., John G.H. (1997). Wrappers for feature subset selection. Artif. Intell..

[39] Abdel-Basset M., Abdel-Fatah L., Sangaiah A.K. (2018). Metaheuristic algorithms: A comprehensive review. Comput. Intell. Multimed. Big Data Cloud Eng. Appl..

[40] Yang X.-S., Yang X.-S. (2021). Chapter 1—Introduction to Algorithms. Nature-Inspired Optimization Algorithms.

[41] Liu W., Wang J. A Brief Survey on Nature-Inspired Metaheuristics for Feature Selection in Classification in this Decade. Proceedings of the 2019 IEEE 16th International Conference on Networking, Sensing and Control (ICNSC).

[42] Agrawal P., Abutarboush H.F., Ganesh T., Mohamed A.W. (2021). Metaheuristic Algorithms on Feature Selection: A Survey of One Decade of Research (2009–2019). IEEE Access.

[43] Faramarzi A., Heidarinejad M., Mirjalili S., Gandomi A.H. (2020). Marine Predators Algorithm: A nature-inspired metaheuristic. Expert Syst. Appl..

[44] Rai R., Dhal K.G., Das A., Ray S. (2023). An Inclusive Survey on Marine Predators Algorithm: Variants and Applications. Arch. Comput. Methods Eng..

[45] Ewees A.A., Ismail F.H., Ghoniem R.M., Gaheen M.A. (2022). Enhanced Marine Predators Algorithm for Solving Global Optimization and Feature Selection Problems. Mathematics.

[46] Zhao W., Wang L., Zhang Z. (2019). Atom search optimization and its application to solve a hydrogeologic parameter estimation problem. Knowl.-Based Syst..

[47] Kamel S., Hamour H., Ahmed M.H., Nasrat L. Atom Search optimization Algorithm for Optimal Radial Distribution System Reconfiguration. Proceedings of the 2019 International Conference on Computer, Control, Electrical, and Electronics Engineering (ICCCEEE).

[48] Bairathi D., Gopalani D. A novel swarm intelligence based optimization method: Harris' hawk optimization. Proceedings of the Intelligent Systems Design and Applications: 18th International Conference on Intelligent Systems Design and Applications (ISDA 2018).

[49] Heidari A.A., Mirjalili S., Faris H., Aljarah I., Mafarja M., Chen H. (2019). Harris hawks optimization: Algorithm and applications. Future Gener. Comput. Syst..

[50] Arora S., Singh S. (2019). Butterfly optimization algorithm: A novel approach for global optimization. Soft Comput..

[51] Zhou H., Cheng H.-Y., Wei Z.-L., Zhao X., Tang A.-D., Xie L. (2021). A Hybrid Butterfly Optimization Algorithm for Numerical Optimization Problems. Comput. Intell. Neurosci..

[52] Mirjalili S., Lewis A. (2016). The whale optimization algorithm. Adv. Eng. Softw..

[53] Mirjalili S., Mirjalili S.M., Lewis A. (2014). Grey wolf optimizer. Adv. Eng. Softw..

[54] Emary E., Zawbaa H.M., Hassanien A.E. (2016). Binary grey wolf optimization approaches for feature selection. Neurocomputing.

[55] Tu Q., Chen X., Liu X. (2019). Multi-strategy ensemble grey wolf optimizer and its application to feature selection. Appl. Soft Comput..

[56] Yang X.-S. (2010). A new metaheuristic bat-inspired algorithm. Nature Inspired Cooperative Strategies for Optimization (NICSO 2010).

[57] Yang X.-S., Yang X.-S. (2021). Chapter 11—Bat Algorithms. Nature-Inspired Optimization Algorithms.

[58] Yang X.-S., He X. (2013). Bat algorithm: Literature review and applications. Int. J. Bio-Inspired Comput..

[59] Yang X.-S. (2010). Firefly algorithm, stochastic test functions and design optimisation. Int. J. Bio-Inspired Comput..

[60] Fister I., Fister Jr I., Yang X.-S., Brest J. (2013). A comprehensive review of firefly algorithms. Swarm Evol. Comput..

[61] Cristianini N., Shawe-Taylor J. (2000). An Introduction to Support Vector Machines and Other Kernel-Based Learning Methods.

[62] Smola A.J., Schölkopf B. (2004). A tutorial on support vector regression. Stat. Comput..

[63] Jun C. (2017). Brain Tumor Dataset. https://figshare.com/articles/dataset/brain_tumor_dataset/1512427.

[64] Cheng J., Huang W., Cao S., Yang R., Yang W., Yun Z., Wang Z., Feng Q. (2015). Enhanced Performance of Brain Tumor Classification via Tumor Region Augmentation and Partition. PLoS ONE.

[65] Rehman A., Naz S., Razzak M.I., Akram F., Imran M. (2020). A Deep Learning-Based Framework for Automatic Brain Tumors Classification Using Transfer Learning. Circuits Syst. Signal Process..

