# Supplementary material for: WBM-DLNets: Wrapper-Based Metaheuristic Deep Learning Networks Feature Optimization for Enhancing Brain Tumor Detection
Source: Bioengineering (Basel). 2023 Apr 14;10(4):475. doi: 10.3390/bioengineering10040475 (PMC10135892; doi:10.3390/bioengineering10040475)
Supplement: Supplementary file 1 [file bioengineering-10-00475-s001.zip › bioengineering-2326008-supplementary.pdf]

Supplementary Materials:

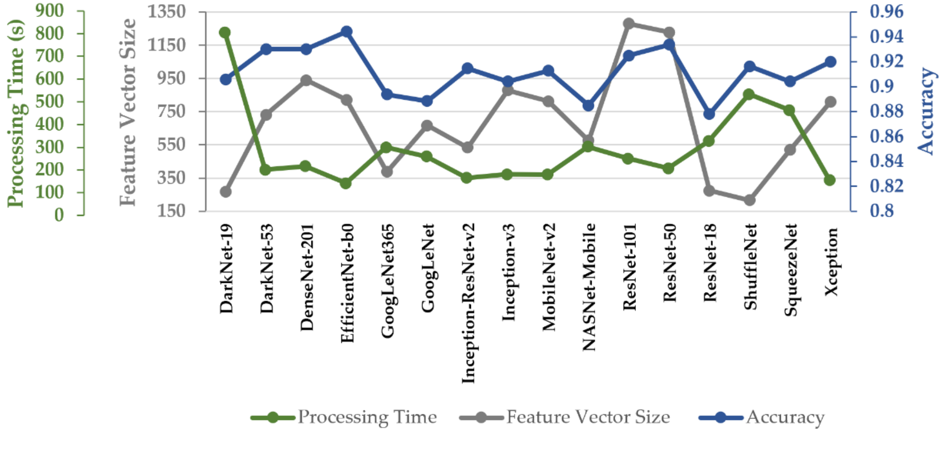

(**a**)

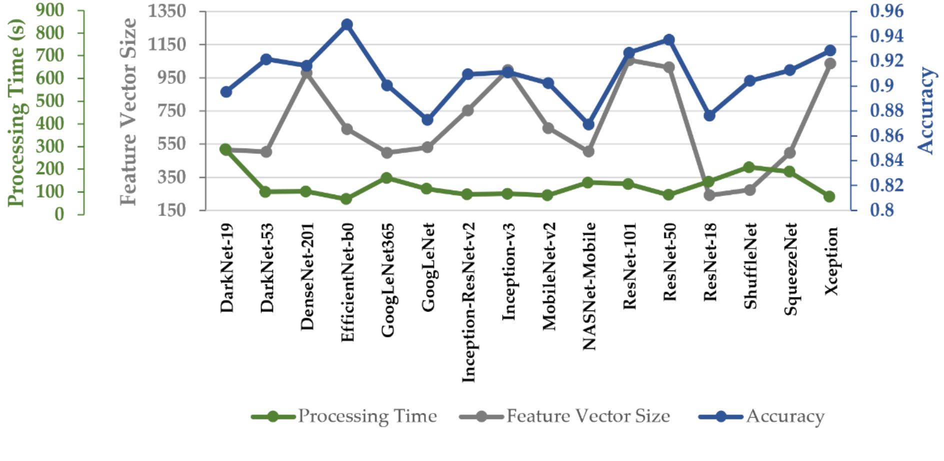

(**b**)

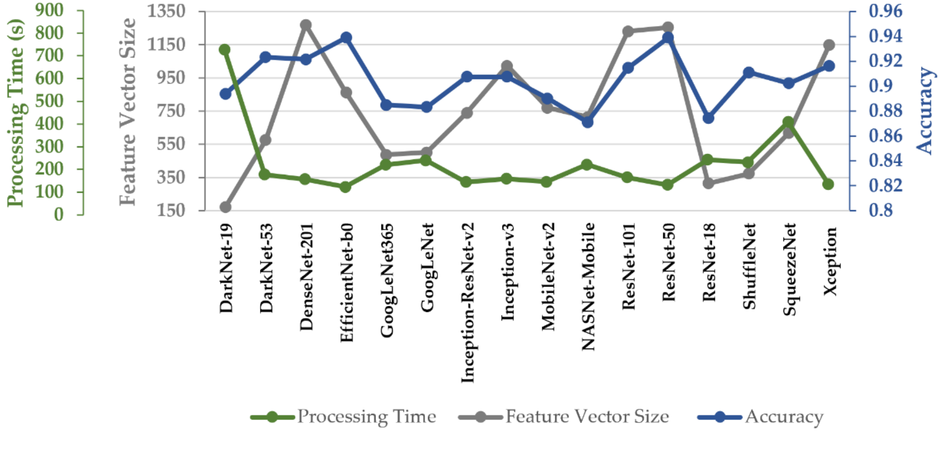

(**c**)

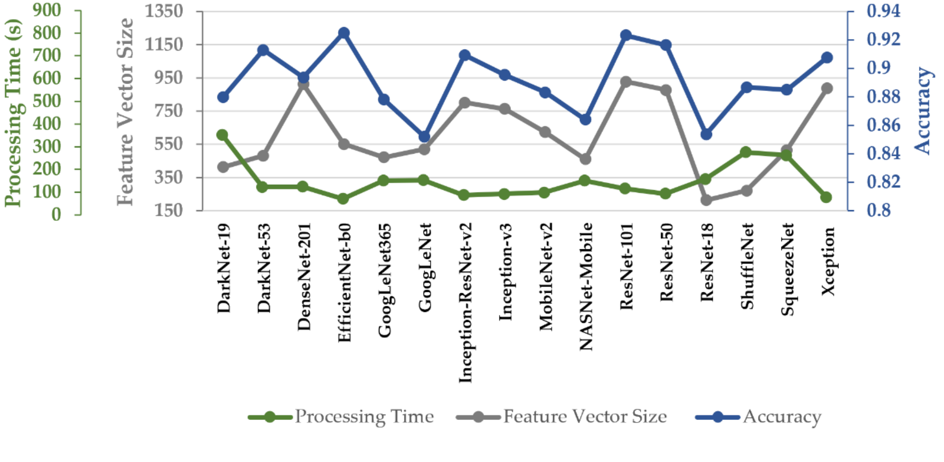

(**d**)

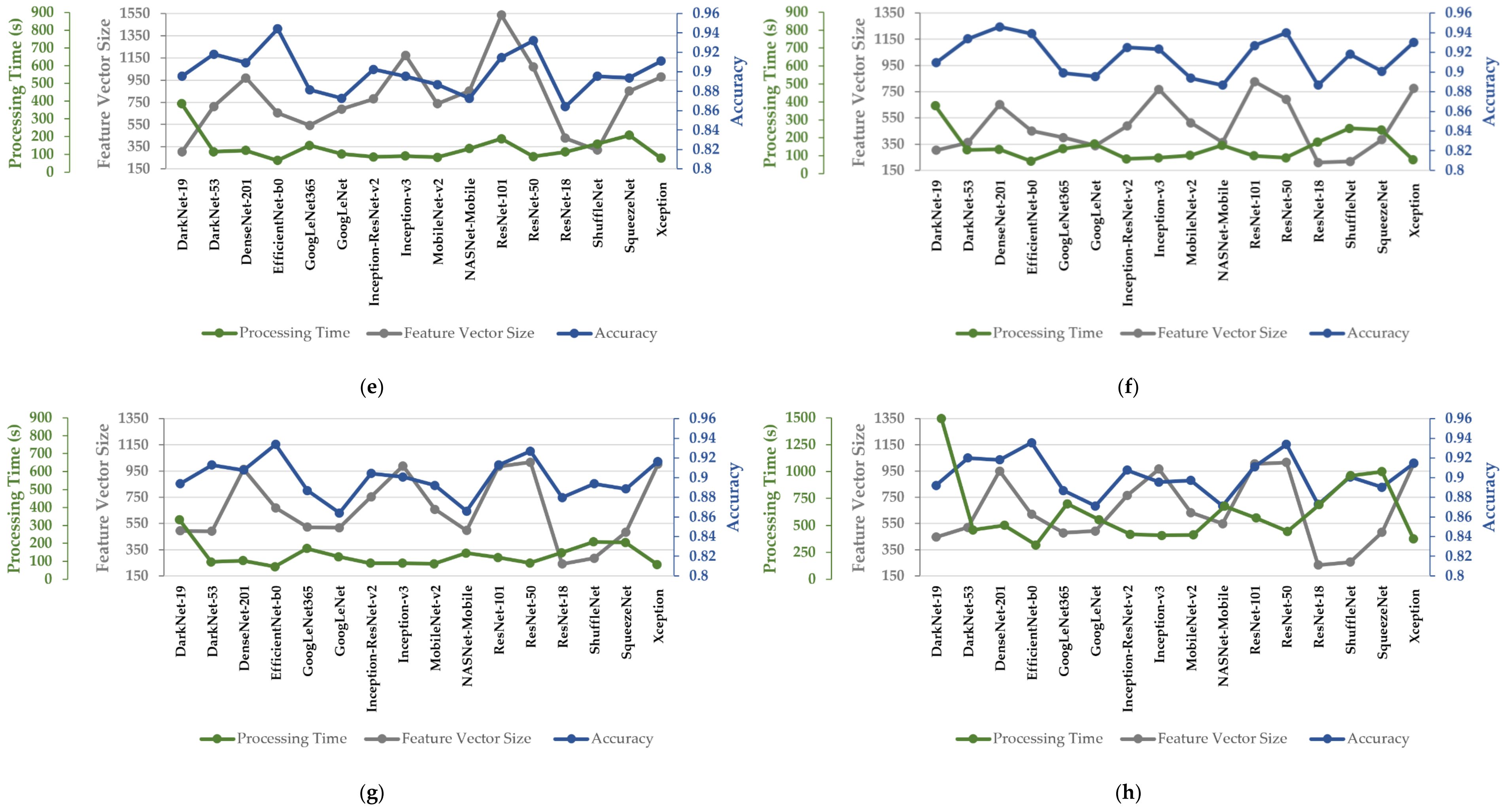

Figure S1. Detailed results of all the networks and the optimization algorithms in terms of accuracy, feature vector size, and processing time: **a)** MPA; **b)** ASOA; **c)** HHOA; **d)** BOA; **e)** WOA; **f)** GWOA; **g)** BA; **h)** FA
